# Changes in cholesterol kinetics following sugar cane policosanol supplementation: a randomized control trial

**DOI:** 10.1186/1476-511X-7-17

**Published:** 2008-04-30

**Authors:** Amira N Kassis, Peter JH Jones

**Affiliations:** 1 School of Dietetics and Human Nutrition, McGill University, Ste-Anne-de-Bellevue, Montréal, Quebec, H9X 3V9, Canada; 2 Richardson Centre for Functional Foods and Nutraceuticals, University of Manitoba, SmartPark, Winnipeg, MB R3T 6C5, Canada

## Abstract

**Background:**

Sugar cane policosanols (SCP) have been shown to exert cholesterol-modulating properties in various studies conducted in Cuba by substantially reducing cholesterol synthesis. Independent research examining changes in cholesterol kinetics in response to SCP is limited to few studies, none of which was able to replicate findings of the original research. Moreover, no data are available on the effect of SCP on cholesterol absorption to date. The present study was undertaken to determine effects on cholesterol kinetics, namely synthesis and absorption, within hypercholesterolemic individuals consuming a SCP treatment. Twenty-one otherwise healthy hypercholesterolemic subjects participated in a randomized double-blind crossover study where they received 10 mg/day of policosanols or a placebo incorporated in margarine as an evening snack for a period of 28 days. The last week of the study phase, subjects were given ^13^C labelled cholesterol and deuterated water for the measurement of cholesterol absorption and synthesis respectively. Blood was collected on the first two and last five days of the trial. Cholesterol absorption and synthesis were determined by measuring red cell cholesterol ^13^C and deuterium enrichment, respectively.

**Results:**

There was no significant change in LDL cholesterol levels as compared to control. In addition, the area under the curve for red cell cholesterol ^13^C enrichment across 96 hours was not significantly different in the SCP group as compared to control. Similarly, no difference was observed in the fractional rate of cholesterol synthesis over the period of 24 hours between the two treatment groups.

**Conclusion:**

The findings of the present study fail to support previous research concerning efficacy and mechanism of action for policosanols.

## Introduction

The most commonly used cholesterol-lowering agents contribute to the prevention of cardiovascular disease through their action on cholesterol metabolism, namely absorption and synthesis [1-3]. Until recently, sugar cane policosanols (SCP) have been extensively researched within a single jurisdiction in Cuba, purporting efficacy in suppressing cholesterol biosynthesis, resulting in up to 30% reductions in plasma low density lipoprotein cholesterol (LDL-C). SCP supplementation studies have established that its cholesterol-lowering effect, mainly mediated by a decrease in serum LDL-C levels, is due to the inhibition of hepatic cholesterol synthesis and stimulation of receptor-mediated LDL uptake by the liver [4-7]. Cholesterol synthesis was assessed *in vitro *by measuring the enrichment of tritiated water into human fibroblasts [7] and animals *in vivo *[6]. In these studies, synthesis was seen to decrease as a result of SCP treatment.

Unlike statins which inhibit cholesterol biosynthesis by directly affecting the rate-limiting enzyme HMG-CoA reductase, SCP seems to have a different, more complex mechanism of action [5]. In human cultured fibroblasts [5,7] using ^14^C acetate and ^14^C mevalonate as tracers, SCP interfered with the cholesterol biosynthesis pathway at a step between acetate and mevalonate production. Since decreased cholesterol biosynthesis is associated with increased LDL-receptor expression in hepatocytes [8,9], SCP effects on serum LDL-C can be suggested as originating from increased LDL- binding, uptake or degradation, as demonstrated in studies measuring radiolabeled LDL on the surface of human fibroblasts [5].

Although studies conducted in Cuba agree internally concerning effects of SCP on cholesterol biosynthesis, only two studies have been conducted outside the Cuban jurisdiction. The first of these two studies, an *in vitro *trial, confirmed findings from the original research [10]. The second trial, conducted on animals, presented conflicting outcomes [11]. Indeed, the latter contributes to the controversy associated with policosanols, showing that cholesterol biosynthesis was not affected in hamsters fed SCP [11]. We thus recognize that the lipid-lowering mechanism of SCP is not fully understood, especially since other cholesterol-lowering mechanisms such as inhibition of cholesterol intestinal absorption have not been investigated.

The objective of the present study was therefore to examine the effect of SCP *in vivo *on cholesterol synthesis and absorption in hypercholesterolemic individuals. We hypothesized that there would be no significant change in cholesterol synthesis and/or absorption as a result of SCP supplementation for 28 days.

## Subjects and methods

### Subjects and treatments

Otherwise healthy hypercholesterolemic men and post-menopausal women (n = 21) age 40 to 80 years, BMI ranging between 23 and 30 kg/m^2 ^were recruited for the clinical trial. Volunteers were asked to visit the research unit for two screenings including a blood draw to determine their lipid profile and other health parameters as well as a medical exam. Subjects accepted in the study had plasma LDL-C levels ranging from 3.0 mmol/l to 5.0 mmol/l and triacylglycerol levels below 4.0 mmol/l at screening.

Exclusion criteria included history of recent or chronic use of oral hypolipidemic therapy, chronic use of insulin, systemic antibodies, corticosteroids, androgens or phenytoin. Subjects were also excluded if they had experienced a myocardial infarction, coronary artery bypass or other major surgical procedures within the last six months, or reported recent onset of angina, congestive heart failure, inflammatory bowel disease, pancreatitis, or hypothyroidism. Significant pre-existing diseases including cancer, chronic use of laxatives as well as smoking or consumption of more than 2 drinks per day were also considered to be part of the exclusion criteria. Before enrolment, subjects were asked to sign a consent form outlining the details of the trial. All procedures included in the protocol were approved by the ethics committee of the medical faculty of McGill University.

Policosanols derived from sugar cane wax (Lipex, Dalmer Laboratories, La Havana, Cuba) were purchased in the form of 5 mg pills which were crushed and incorporated into margarine. The composition of the SCP treatment was: 65.6% octacosanol, 13.4% triacontanol and 4.5% hexacosanol [12]. The daily dose of treatment provided 10 mg of policosanols mixed with 10 g of margarine (Becel, Canada) and was served on a slice of bread. [3,4- ^13^C] cholesterol and deuterium oxide (99.8 atom percent excess) were obtained from CDN isotopes (Montreal, Canada). [3,4- ^13^C] cholesterol was incorporated in margarine (Becel, Canada) by thorough mixing at a concentration of 7.5 mg/g margarine.

### Protocol

The study was designed as a randomized double-blind crossover where subjects were assigned to receive the SCP or placebo margarines as a daily evening snack for a period of 28 days. The two treatment phases were separated by a wash out period of the same duration. Study participants were asked to visit the clinic everyday and consume the snacks under staff supervision at the Mary Emily Clinical Research Centre, in order to ensure absolute compliance. Subjects were requested to maintain their habitual diet and physical activity patterns throughout the study and to abstain from alcohol throughout the treatment phases. Caffeinated drinks were restricted to one cup per day and subjects were offered caffeine-free beverages with their snacks. At the beginning and end of each phase, a three-day 24-hour recall was recorded for each subject to ensure that there were no substantial changes in their dietary habits within and between the two phases. Body weights were recorded daily in order to monitor weight fluctuations throughout the study period.

On day 25, subjects were administered 10 g of margarine spread on a slice of bread providing a dose of 75 mg of [3,4- ^13^C] cholesterol. Blood was collected before isotope ingestion (hour 0) and 24, 48, 72 and 96 hours after ingestion in order to measure cholesterol absorption. On day 28, subjects were asked to drink a dose of 0.7 g deuterium oxide/kg body water (60% of body weight recorded during the last 5 days). Blood was collected before isotope administration (hour 0) and at hour 24 for cholesterol biosynthesis measurements.

### Nutrient composition of the diet

Subjects' food intake for the previous day was recorded at the start and end of each intervention phase. Subjects were advised to write the food items and the exact quantity after each meal to reduce the memory bias during the interview the next day. They were provided with portion models, including cups, plates, bowls, spoons to help them report portions more accurately. Nutrient composition of food items was analyzed using Food Processor version 1.0 and the average macronutrient, cholesterol and dietary fibre content of the diet over three days was computed at the start and end of the phase. Macronutrient intake was then expressed as percent energy.

### Determination of cholesterol absorption

Blood was collected by venipuncture into 10 ml EDTA tubes. Red blood cells (RBCs) were separated from plasma by centrifugation at 15,000 rpm for 30 minutes and stored at -80°C for further analysis. Cholesterol absorption was measured using the single-stable isotope-labeled cholesterol tracer approach which was validated against the commonly used dual stable-isotope ratio method and shown to be reliable in the determination of cholesterol intestinal absorption [13]. The lipid fraction of RBCs was isolated using the modified Folch extraction procedure [14]. Lipid extracts were analyzed using gas chromatography isotope ratio mass spectromety (GC-IRMS, ThermoFinnigan, Bremen, Germany) where samples were run through a GC unit, a combustion reactor and a mass spectrometer. Briefly, lipids were separated by gas chromatography using a SAC-5 sterol column (Supelco, Milton, Ontario) and isolated cholesterol was directed to the combustion reactor to release CO_2 _into the MS where ^13^C enrichment of CO_2 _(δ ^13^CO2/^12^CO_2_) was determined relative to the reference gas and expressed relative to the international standard Vienna Pee Dee Belemita (V-PDB) limestone. Enrichments were measured for each of hours 0, 24, 48, 72 and 96 and the area under the curve (AUC) was used to compare cholesterol absorption in the SCP and control groups.

### Determination of cholesterol biosynthesis

Blood was collected on day 28 (hour 0) and 29 (hour 24) and lipid extraction performed using the same methods mentioned for the determination of cholesterol absorption. Cholesterol synthesis was assessed by measuring the rate of deuterium incorporation from body water into RBC membrane free cholesterol over 24 hours. Lipid extracts from RBCs were separated using GC and isolated cholesterol was introduced into a pyrolysis reactor to release H_2 _gas. Plasma water samples were run through a high temperature conversion elemental analyzer (TC-EA). Deuterium enrichments for both RBCs and plasma water were measured by IRMS relative to the reference gas. Normalization to Vienna standard mean ocean water (V-SMOW) was performed using a regression equation between the online and offline method (as mentioned above) with data from the offline method expressed relative to V-SMOW. The fractional rate of synthesis (FSR) for cholesterol was then calculated using the following equation:

FSR (pools/day) = (δ C/δ PW) × 0.478

Where:

δ C = difference in deuterium enrichment between hour 0 and hour 24 for cholesterol

δ PW = difference in deuterium enrichment between hour 0 and hour 24 for plasma water

0.478 = ratio of labeled H atoms replaced by deuterium during *in vivo *biosynthesis

### Statistical analysis

The sample size (n = 21) for the clinical trial was calculated to provide an 80% probability of detecting an anticipated difference in parameters tested of 20%, using a coefficient of variation of 15–20%. Effects of SCP treatment on cholesterol absorption and synthesis were compared to control using a one-way ANCOVA for crossover models. The effect of sequence, treatment, period, subject and gender were included in the model. Macronutrient, cholesterol and dietary fibre intakes were compared at the start and end of each phase and between the two interventions using student t-tests. Multiple regressions between cholesterol kinetic parameters (AUC and FSR) and nutrient intake as well as age, BMI and LDL levels were performed to identify potential covariates. The model was subsequently adjusted for those covariates. Statistical significance was set at p < 0.05.

## Results

### Compliance and drop-out rate

Twenty-two subjects were recruited to take part in the clinical trial and twenty-one successfully completed the study. Baseline characteristics of subjects are presented in Table 1. The SCP and placebo margarines were well-tolerated. No serious adverse effects were reported throughout the trial with the exception of four subjects reporting gastrointestinal discomfort and more frequent bowel movements. Two of these were in the SCP group while the other two received the control intervention. Body weights remained stable throughout the study periods and blood biochemistry and hematology were within normal ranges.

**Table 1:** Baseline characteristics of subjects (n = 21)

**Variable**	**Males (n = 12)**	**Females (n = 9)**	**All (n = 21)**
Age (y)	54.0 ± 2.9 ^a^	60.1 ± 2.6^a^	57.8 ± 2.1
Weight (kg)	84.8 ± 4.9 ^a^	71.1 ± 3.8 ^b^	76.3 ± 3.2
BMI (kg/m^2^)	26.9 ± 1.0 ^a^	26.3 ± 0.8 ^a^	26.5 ± 0.6
Total cholesterol (mmol/l)	5.4 ± 0.4 ^a^	6.09 ± 0.3 ^a^	5.8 ± 0.2
LDL- cholesterol (mmol/l)	3.5 ± 0.3 ^a^	3.8 ± 0.3 ^a^	3.7 ± 0.2
HDL- cholesterol (mmol/l)	1.2 ± 0.1 ^a^	1.6 ± 0.1 ^b^	1.4 ± 0.09
Triacylglycerols (mmol/l)	1.5 ± 0.4 ^a^	1.7 ± 0.3 ^a^	1.6 ± 0.2

Values with different superscript letters are statistically different at p < 0.05.

Male/Female ratio = 1.3

### Plasma lipid levels

Plasma lipid levels were measured and results were published in previous work [15]. Briefly, no significant differences were observed between SCP treatment and control groups in plasma total, LDL, HDL and triacylglycerol levels. These outcomes were observed both with endpoint values and percent changes in blood lipids.

### Dietary intake throughout the study period

Macronutrient, cholesterol and dietary fiber intakes throughout the study are presented in Table 2. Values are presented as mean ± SEM. Nutrient content and composition of the subjects' diet were similar at the beginning and end of the two intervention phases. Average caloric and nutrient intakes throughout the SCP intervention were 2005 ± 127 calories, 35.7% from fat (10.9% saturated), 15.1% from proteins and 51.1% from carbohydrates. In comparison, subjects consumed 2109 ± 127 calories, 35.5% from fat (11.0% saturated), 15.3% from proteins and 50.4% from carbohydrates. Subjects' dietary cholesterol and fibre intakes were assessed as 141.3 ± 12.7 mg/day and 19.9 ± 1.6 g/day respectively during the SCP intervention. Subjects in the control intervention had cholesterol and dietary fibre intakes of 120.5 ± 12.7 mg/day and 20.0 ± 1.6 g/day respectively. No differences between groups were noted for all nutrients demonstrating that study participants maintained their dietary habits throughout the study period.

**Table 2:** Nutrient composition of subjects' diet throughout the study period (n = 21)

	Control		SCP	
Average intake/day	Start	End	Start	End
Energy (Kcal)	2126.2 ± 152.0	2084.5 ± 148.3	1900.0 ± 130.2	2109.8 ± 130.2
Fat (g)	87.03 ± 6.92	80.41 ± 6.76	72.71 ± 5.05	83.77 ± 5.05
% energy from fat	37.15 ± 1.66	34.26 ± 1.62	35.33 ± 1.41	36.15 ± 1.41
Saturated fat (g)	26.37 ± 2.32	25.09 ± 2.26	22.97 ± 1.94	24.84 ± 1.94
Energy from saturated fat (%)	11.33 ± 0.69	10.84 ± 0.67	11.10 ± 0.66	10.70 ± 0.66
Proteins (g)	75.90 ± 6.46	82.47 ± 6.31	68.52 ± 4.30	79.23 ± 4.30
Energy from proteins (%)	14.59 ± 0.80	15.91 ± 0.78	14.68 ± 0.68	15.47 ± 0.68
Carbohydrates (g)	270.29 ± 22.3	262.59 ± 21.7	252.82 ± 22.4	269.33 ± 22.4
Energy from carbohydrates (%)	50.28 ± 1.65	50.36 ± 1.61	52.11 ± 1.77	50.11 ± 1.77
Cholesterol (mg)	261.14 ± 32.8	249.95 ± 32.0	249.64 ± 33.9	304.50 ± 33.9
Cholesterol (mg/1000 Kcal)	123.95 ± 11.1	116.70 ± 10.8	130.84 ± 7.21	151.65 ± 17.2
Dietary fibre (g)	20.98 ± 1.77	18.82 ± 1.73	19.34 ± 1.79	20.71 ± 1.79

### Cholesterol absorption as a result of SCP supplementation

**Figure 1:**
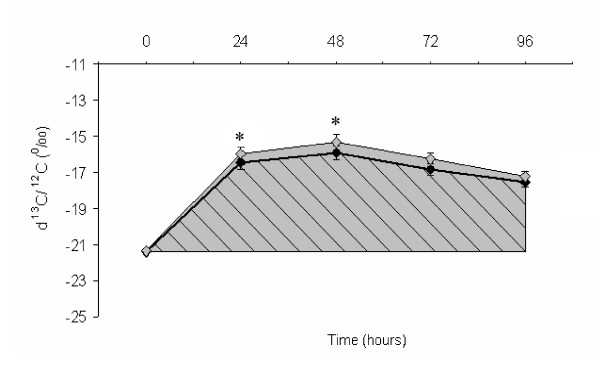
**^13^C enrichment of RBCs after isotope administration in SCP and control groups (n = 21)**. Black diamond: sugar cane policosanols, grey diamond: placebo. AUC _SCP _= 421.91; AUC_control _= 454.55. No significant difference between the two intervention groups. n = 21.

**Figure 2:**
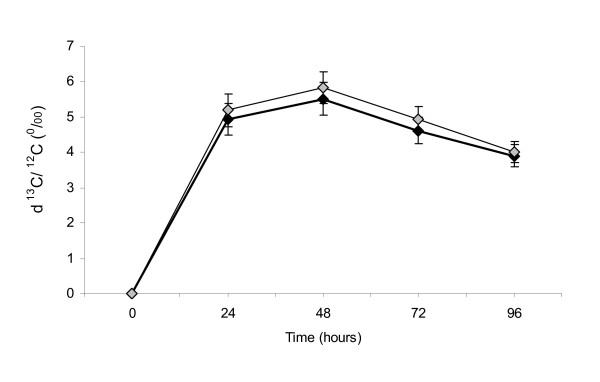
**Difference in ^13^C enrichment between time points and hour 0 as a result of isotope administration in SCP and control groups (n = 21)**. Black diamond: sugar cane policosanols, grey diamond: placebo. No significant difference between SCP and control groups. n = 21.

Cholesterol ^13 ^C enrichments in red blood cells at different time points are illustrated in Figure 1. Values are presented as mean ± SEM. At hour 24, recorded enrichments versus V-PDB were (p = 0.0098) -16.4 ± 0.43 ‰ and -16.0 ± 0.43 ‰ in the SCP and control groups, respectively. At hour 48, enrichment values in the two groups were (p = 0.0221) -15.9 ± 0.42 ‰ and -15.3 ± 0.43 ‰ respectively. At other time points, no significant difference was observed between SCP and control treatments. When enrichment values were calculated as the difference from baseline (Figure 2), SCP supplementation showed no significant effect on this parameter at all time points. Cholesterol absorption rate was determined as the area under the ^13^C enrichment curve. Calculated AUC for SCP and control is presented in Figure 1. Results show that in the SCP group, cholesterol absorption was lower than control by 7.2%, although the difference did not reach significance (p = 0.10). The fixed effect of gender on cholesterol absorption was significant (p = 0.03), and was manifested by a mean AUC of 513 in female subjects as compared to 362 in males. The effect of diet as determined by multiple regressions was significant (p = 0.02) for caloric intake which was included in the model as a covariate. Adjusted AUC means in the two interventions were not significantly different (p = 0.1).

### Cholesterol synthesis as a result of SCP supplementation

Cholesterol fractional rate of synthesis was (mean ± SEM) 0.0169 ± 0.001 pools/day and 0.0170 ± 0.001 pools/day in the SCP and control groups respectively with no significant difference between the effects of the two interventions. Results show no difference in FSR between males and females (p = 0.65). Daily intake of energy (p = 0.03) and energy from saturated fat (p = 0.03) had significant effects on FSR and were included in the model as covariates. The effect of SCP treatment remained non-significant after adjustment for covariates.

## Discussion

The present study is the first human clinical trial to assess the effect of SCP on cholesterol intestinal absorption and biosynthesis using cholesterol isotope labeling methodologies. Our results do not agree with the larger body of evidence showing that SCP interfere with the biosynthetic pathway of cholesterol in the liver decreasing its levels in the blood in either animals [4,6] and humans [5,16,17]. The research group originating these claims used rats and rabbits treated with doses of SCP and measured the rate of incorporation of ^3^H from tritium oxide into hepatic sterols [4,6]. The authors of both animal trials report significant reductions of tritium incorporation of up to 50% as a result of SCP administration to the animals concluding that SCP reduced the rate of cholesterol biosynthesis. However, these findings were challenged by more recent animal data [11] using deuterium incorporation into red blood cells in a hamster model treated with SCP and showing no effect on cholesterol biosynthesis as a result of SCP administration. In another trial from the same authors, different doses of policosanols were seen to increase the rate of cholesterol biosynthesis in the animals [18]. In humans, no studies are available that examine the effect of SCP on cholesterol metabolism *in vivo*. Rather, *in vitro *experiments were conducted on human fibroblasts [5,16,17] and hepatoma cells [10] incubated with increasing doses of policosanols and subjected to the addition of ^14^C-acetate and ^14^C-mevalonate. A clear dose-dependant reduction was observed in the incorporation of ^14^C-acetate into cellular cholesterol as a result of SCP treatment [5,10]. In fact, as policosanol concentration increased, radioactivity incorporated from acetate, but not from mevalonate, decreased significantly reaching an inhibition of 30% [10] and 49.7% [5] at the 25 μg/ml and 50 μg/ml dose respectively. The authors concluded that policosanols interfered in the cholesterol synthetic pathway at a step between acetate and mevalonate production.

While the evidence from these experiments seems compelling, the method of choice can be strongly criticized when applied to metabolically active compounds, in this case policosanols. In a cellular medium, policosanols are metabolized into their corresponding acids and subsequently enter the beta-oxidation pathway [19]. A dilution effect can be suggested in this case whereby policosanol-derived acetate contributes to reducing the ratio of ^14^C/^12^C-acetate in the medium, resulting in an artificial inhibitory effect of policosanols on cholesterol synthesis. This hypothesis would explain the absence of effect when using ^14^C-mevalonate which is not a product of beta oxidation of policosanols. The present study overcomes the aforementioned limitations by examining the effect of SCP *in vivo*, using deuterium incorporation into red blood cell cholesterol. This method has been used in numerous human trials and was shown to be an accurate, reliable and safe method for the determination of cholesterol synthesis [20-23].

The study was designed so that subject compliance to the protocol was ensured by close supervision from the clinical staff and that the SCP treatment was the one used in earlier original research at a dose previously shown to decrease cholesterol levels in humans [24-26]. Moreover, the treatment was administered in the evening because cholesterol synthesis has been shown to reach maximal values at night [27,28]. It remains that no significance was recorded as to the effect of SCP on cholesterol biosynthesis which explains the inefficacy of the treatment in improving the lipid profile of study participants and supports the growing body of evidence originating from independent research groups reporting no change in blood lipids [15,29-33].

The poor absorption of SCP in the gut and the increase in synthesis as a result of treatment seen in the Wang et al study [18] generated the interest in determining the effect of SCP on cholesterol absorption in the present study. A mechanism of action similar to that of plant sterols whereby SCP would compete with cholesterol in the gut [34], decreasing its absorption and slightly increasing its synthesis, was suggested as a hypothesis in this study. Although absolute ^13^C enrichments were significantly lower than control in the SCP group at hour 24 and 48, the effect disappeared when levels were adjusted for baseline. Moreover, the AUC for ^13^C enrichments, a better marker for cholesterol absorption was not significantly different across intervention groups. Results thus show no effect of SCP on cholesterol absorption in the gut, and this independently of the subjects' dietary intake.

Earlier human efficacy studies on SCP did not control dietary intake of participants [35-38]. Subjects were given dietary advice and asked to follow an NCEP step I diet. In the present study, subjects were asked to maintain a stable diet and food intake was recorded thoroughly in order to ensure there were no nutritional differences between the two intervention groups. Results show that nutrient intake remained stable throughout the study phases and between the two groups allowing us to rule out the possibility of a confounding effect of food. Nutrient analyses also showed that caloric intake was negatively related to cholesterol absorption and positively to synthesis. A similar relationship has been reported previously in trials looking at caloric restriction, weight loss and their effect on cholesterol synthesis [39-41], however, few studies have examined the impact of caloric restriction on cholesterol absorption. Although two studies including diabetic subjects observed a negative relationship between cholesterol absorption and caloric intake [42,43], a more recent trial has shown no significant effect of caloric restriction or weight loss on cholesterol absorption [41]. Percent energy from saturated fat in the subjects' diet was negatively related to cholesterol synthesis across intervention groups. Saturated fats have long been associated with increased blood cholesterol [44,45], however, their effect on cholesterol metabolism depends on other factors such as concurrent intake of dietary cholesterol and the ratio of polyunsaturated to saturated fats [46]. Since dietary intake and fatty acid composition were not controlled in this study, the relationship between saturated fat and cholesterol metabolism remains inconclusive. When energy intake and percent saturated fat were adjusted for in the model as covariates, the SCP treatment remained ineffective in altering parameters of cholesterol metabolism.

Considering previous independent findings on the inefficacy of SCP [15,29,32,47] as well as its poor absorption [12,19], the claim that SCP inhibits cholesterol synthesis by an indirect effect on HMG-CoA reductase in peroxisomes is further refuted by the outcomes of the present study. We therefore conclude that the natural lipid-lowering agent does not alter cholesterol intestinal absorption and/or hepatic biosynthesis.

## Competing interests

The authors Amira N. Kassis and Peter J.H. Jones have no conflicts of interest with the present work

## Authors' contributions

AK was involved in the study design, ran the clinical trial collected and analyzed data and wrote the manuscript. PJ designed the study protocol and offered supervision and guidance during data collection and manuscript writing. All authors have read and approved the final manuscript.
